# F60. INFLAMMATORY MARKERS AND COGNITIVE PERFORMANCE IN PATIENTS WITH SCHIZOPHRENIA TREATED WITH LURASIDONE

**DOI:** 10.1093/schbul/sby017.591

**Published:** 2018-04-01

**Authors:** Andrei Pikalov, Brian Miller, Cynthia Siu, Michael Tocco, Joyce Tsai, Philip Harvey, Antony Loebel

**Affiliations:** 1 Sunovion Pharmaceuticals, Inc; 2 Medical College of Georgia, Georgia Regents University; 3 COS and Associates Ltd., Central District; 5 Leonard M. Miller School of Medicine, University of Miami

## Abstract

**Background:**

Recent studies have linked inflammation, obesity, and lipid dysregulation with cognitive impairment, a core feature of schizophrenia. Elevated C-reactive protein concentration has been shown to be a reliable biomarker for inflammatory states. We conducted an exploratory analysis to investigate the potential influence of inflammation, obesity and lipid metabolism on changes in symptom severity and cognitive performance in patients with schizophrenia treated with lurasidone.

**Methods:**

Patients with acute exacerbation of schizophrenia were treated with one of two fixed doses of lurasidone (80 or 160 mg/day), placebo, or 600 mg/day quetiapine XR in a 6-week double-blind study. A wide-range CRP (wr-CRP) assay (equivalent to high sensitivity CRP assay) was used to assess levels of inflammation. CRP was evaluated as a logarithm transformed (log) continuous variable and as a categorical variable divided into low (≤ 2 mg/L), medium (> 2 mg/L and ≤ 5 mg/L) and high (> 5 mg/L) subgroups. Cognitive function was assessed with the CogState computerized cognitive battery at baseline and week 6 endpoint. Nonparametric bootstrap resampling method was applied to estimate the main and interactive effects of CRP on ranked cognitive scores.

**Results:**

Elevated level of wr-CRP (log) was associated with cognitive impairment at study baseline (P < 0.05), with significantly lower cognitive performance in the subgroup with high wr-CRP (> 5 mg/L) compared to those with low wr- CRP (< 2 mg/L) at study baseline (P < 0.05). Higher level of CRP (log) was also associated with significantly greater symptom severity as assessed by PANSS score, as well as higher BMI/body weight, and lower levels of high-density lipoprotein (HDL) and high hemoglobin A1c (HbA1c) at study baseline (P < 0.05). No significant associations were found for wr-CRP (log) with low-density lipoprotein (LDL) and total cholesterol at study baseline. High wr-CRP level (> 5 mg/L) at study baseline predicted less improvement of cognitive composite score at week 6 endpoint for all treatment groups, compared to those with low to medium wr-CRP levels (< 5 mg/L).

The joint effect of wr-CRP (log) and HDL or HOMA-IR on moderating procognitive effects of lurasidone was significant (P<0.05), with greater lurasidone (vs. placebo) effect size in patients with either low wr-CRP and high HDL concentration or lower levels of both wr-CRP and HOMA-IR.

Lurasidone treatment was associated with significant reduction in symptom severity as assessed by PANSS, CGI-S, and MADRS change scores from baseline to week 6, independent of wr-CRP, HDL and HOMA-IR levels at study baseline. Lurasidone had no significant effect on change in wr-CRP level from baseline to week 6 endpoint.

**Discussion:**

Our findings from this exploratory analysis of a placebo-controlled trial in patients with schizophrenia suggest that the joint effects of low wr-CRP level combined with either high HDL or low HOMA-IR can predict cognitive improvement in patients treated with lurasidone (vs. placebo). These findings suggest that inflammation and its interactive effects with insulin resistance and lipid parameters in patients with schizophrenia might impact cognition and response to treatment with antipsychotics.

